# Discrete versus multiple word displays: a re-analysis of studies comparing dyslexic and typically developing children

**DOI:** 10.3389/fpsyg.2015.01530

**Published:** 2015-10-07

**Authors:** Pierluigi Zoccolotti, Maria De Luca, Donatella Spinelli

**Affiliations:** ^1^Department of Psychology, Sapienza University of RomeRome, Italy; ^2^Neuropsychology Unit, Istituto di Ricovero e Cura a Carattere Scientifico – Fondazione Santa LuciaRome, Italy; ^3^Department of Human Movement Sciences and Health, University of Rome “Foro Italico”Rome, Italy

**Keywords:** reading, dyslexia, text reading, multiple displays, vocal reaction time

## Abstract

The study examines whether impairments in reading a text can be explained by a deficit in word decoding or an additional deficit in the processes governing the integration of reading subcomponents (including eye movement programming and pronunciation) should also be postulated. We report a re-analysis of data from eleven previous experiments conducted in our lab where the reading performance on single, discrete word displays as well multiple displays (texts, and in few cases also word lists) was investigated in groups of dyslexic children and typically developing readers. The analysis focuses on measures of time and not accuracy. Across experiments, dyslexic children are slower and more variable than typically developing readers in reading texts as well as vocal reaction time (RTs) to singly presented words; the dis-homogeneity in variability between groups points to the inappropriateness of standard measures of size effect (such as Cohen’s *d*), and suggests the use of the ratio between groups’ performance. The mean ratio for text reading is 1.95 across experiments. Mean ratio for vocal RTs for singly presented words is considerably smaller (1.52). Furthermore, this latter value is probably an overestimation as considering total reading times (i.e., a measure including also the pronunciation component) considerably reduces the group difference in vocal RTs (1.19 according to Martelli et al., 2014). The ratio difference between single and multiple displays does not depend upon the presence of a semantic context in the case of texts as large ratios are also observed with lists of unrelated words (though studies testing this aspect were few). We conclude that, if care is taken in using appropriate comparisons, the deficit in reading texts or lists of words is appreciably greater than that revealed with discrete word presentations. Thus, reading multiple stimuli present a specific, additional challenge to dyslexic children indicating that models of reading should incorporate this aspect.

## Introduction

Reading a passage is a complex task requiring a number of sub-componential tasks, which start from the perception of visual features (contours, segments of various orientations), then letters, and word recognition to continue with the integration of successive words into a coherent stream. At this level, syntactic and semantic processing allows for the identification of the sentence meaning and the possibility to place it within the more general context of the text. All this takes place in association with motor processing, i.e., saccades and fixations to scan the text, and pronunciation. In reading deficiencies, it is interesting to understand which is the level of analysis which is most appropriate to describe the reading difficulty (here, we restrict our analysis to developmental deficits, i.e., developmental dyslexia DD). Potentially, any of the above listed levels may generate the difficulty as research has clearly shown they are all necessary steps in the reading process. So, one may think that the deficit in DD originates as early in the information processing chain as in the elaboration of letters; alternatively, one may see the deficit originating at a word locus or later when the identification of several words is merged as it occurs in the reading of meaningful texts. Note that early deficits (including also motor processing such as eye movements) may spread into later processing as a cascade effect. As an example, if we imagine a child to be impaired in letter recognition (or in the programming of eye movements) this will severely affect all subsequent processing, including word recognition, integration of decoding, and pronunciation etc.

So, one very general question, which has been extensively examined in the literature on DD, is which is the earliest level of processing at which a deficit can be reliably found. It is generally held that children with dyslexia are spared in processing letters. Importantly, evidence is based on a variety of sensitive techniques (such as contrast thresholds, or masked tachistoscopic presentation) that guarantee that this sparing is not due to the lack of sensitivity of the measures used (Bosse et al., 2007; Lassus-Sangosse et al., 2008; Martelli et al., 2009; De Luca et al., 2010). By contrast, it is well established that children with dyslexia are selectively impaired in processing strings of letters (whether forming existing words or not). Indeed, major models of reading (such as the dual route cascaded model or DRC; Coltheart et al., 2001; the CDP+ model; Perry et al., 2007; and the triangle model, Plaut et al., 1996) are focused in explaining reading at the word level. So, up to date evidence indicates that the nuclear deficit in DD is at the level of letter orthographic string decoding.

However, there is reason to think that the reading deficit may not be entirely explained at the word level and that the need to integrate the processing of words with other sub-components of reading may represent an additional burden, which selectively affects the reading of dyslexic children. So, a second general question is whether impairments at subsequent levels of processing can be identified and explained either as independent defects or due to a cascade effect from deficits in orthographic decoding.

Critical to answer this question is the comparison between single, discrete word displays (typical of experimental settings) and multiple displays (as it occurs in the reading of meaningful texts). However, comparing such different levels of processing may prove difficult, *in primis* due to variations in general difficulty of the two tasks. A further difficulty is that different measures are typically used. When single words are examined a frequently used measure is vocal reaction time (RT), i.e., the time between the stimulus onset and the beginning of subject’s vocal response. When texts or lists of words are examined the reading time also includes the time required to utter the sentences (or the words in the list).

Therefore, examining total reading times (i.e., RTs plus pronunciation times) also in the case of singly presented words may be instrumental to compare reading fluency between discrete and multiple displays. In one such study, we observed that typically developing readers showed an advantage on multiple with respect to discrete items: they were able to process the next stimulus while uttering the current word indicating that pronunciation times overlapped with decoding times (Zoccolotti et al., 2013). By contrast, children with dyslexia did not show the advantage for multiple over discrete stimuli in the case of lists of short words and actually showed a disadvantage in the case of long words (on which they were slower than in the case of discrete stimuli). We proposed that the disproportionate impairment of children with dyslexia in dealing with multiple arrays indicates a difficulty in integrating the multiple subcomponents of the reading task over and above the basic nuclear deficit in decoding words (Zoccolotti et al., 2013).

Can we re-evaluate the previous literature in light of the findings indicating a specific deficit in reading sub-components in dyslexia? The main question of the present study is whether impairments in functional reading can be explained by the basic deficit in letter string decoding or an additional deficit in the integration of various reading subcomponent should also be postulated. To this aim we report a re-analysis of data from previous experiments conducted in our laboratory where the reading performance on both single, discrete word and multiple words has been investigated in groups of typically developing and dyslexic children. The analysis focuses on measures of time and not accuracy.

Our first question is whether the reading deficit shown by children with dyslexia is greater with discrete or multiple visual displays. Clearly, the experimental conditions used in our previous studies are not ideal for this comparison. On the one hand, studies on single words typically reported RTs not reading times (i.e., a measure including pronunciation, as in Zoccolotti et al., 2013); thus, one should ideally control for the effect of pronunciation on the results of previous studies. On the other hand, single and multiple stimuli were not matched in terms of stimulus characteristics. Studies based on single word presentation usually aimed to understand the effect on vocal RTs of parameters such as word frequency, word length, morphological structure and so on, often leading to a large number of levels of the experimental manipulations. By contrast, multiple word displays were texts or list of words; these materials are typically used to select the groups of dyslexic and typically developing children according to their basic reading skills and often yield a single measure of overall performance. Thus, to compare the efficiency in reading words in multiple and single stimulus displays we have to average data collected over different experimental conditions in discrete word studies to obtain an overall estimate of the reading time also for singly presented words.

Additional methodological questions arise in the case of such comparison. Namely, which is the appropriate index to compare the size of the difference between dyslexic and control readers? How can the difference in dependent measure (RTs versus total reading times) be controlled for? Does the presence of a meaningful context modulate the performance of children with dyslexia? The way we tackle each of these questions is detailed below along with the presentation of results.

## Methods

### Selection Criteria of Target Studies

We focused on studies in which children with dyslexia were compared to a group of typically developing readers using very similar (although not identical) subject’s selection criteria. We also limited the analysis to groups of children attending sixth grade, which was the most common age in our previous studies. With these criteria we were able to trace eleven different studies where we had both measures of text reading (used for the purpose of screening by standard reading text) and measures of vocal RTs to single word (used for the specific aims of the given study). All but one recently completed study have been previously published. Some of these studies also included different screening tests requiring the reading of lists of words (see below for more details).

### Reading Measures

The basic reading test used for screening purposes was the *MT Reading Test* (Cornoldi and Colpo, 1995): a passage adapted for children’s age is presented and the child is requested to read it as fast and accurately as possible. Two tests requiring the reading of lists of words were used. One was the *Words and Non-words Reading Test* (Zoccolotti et al., 2005). This features four lists of 30 words varying for frequency and length; separate norms are available for each of the four sub-lists. Another test was the word sub-test from the *Battery for the Evaluation of Developmental Dyslexia and Dysgraphia* (Sartori et al., 1995). A total of 112 words are presented in four 28-word sub-lists varying for frequency and imageability. However, only a single measure is usually reported for this test as norms report only this measure of general performance. In both tests, the list of words was printed vertically; the task, as in the *MT Reading Test* and vocal RTs, was to read the words as fast and accurately as possible.

Reaction times were measured in all studies by presenting a word in the center of a computer screen; the word was visible until the children started his/her uttering. The RT was measured as the interval between stimulus onset and vocal onset.

## Results

### Fluency Differences in Reading Texts

**Table 1** presents the list of studies selected, indicating the number of dyslexic children and chronologically matched typically developing children considered in each of them. A total of 331 typically developing children and 172 cases participated to the studies. The mean times for reading a standard text passage (*MT Reading Test*; Cornoldi and Colpo, 1995) are reported for both groups. The mean reading times are expressed in terms of s per word (by averaging words of different length in the paragraph). Various observations can be advanced based on the data in the table.

**Table 1:** Reading times at the MT Reading test.

Reference	Typically developing children				Children with dyslexia				Groups’ performance ratio
	*N*	Mean	*SD*	Coefficient of variation	*N*	Mean	*SD*	Coefficient of variation	
Judica et al., 2002	16	0.62	0.25	0.40	18	1.51	0.82	0.54	2.4
Spinelli et al., 2005	23	0.56	0.14	0.25	17	1.10	0.42	0.38	2.0
Barca et al., 2006	68	0.55	0,10	0,18	14	0.94	0,26	0,28	1.7
Zoccolotti et al., 2006	12	0.52	0.09	0.18	10	1.10	0.48	0.43	2.1
Burani et al., 2008	34	0.56	0.09	0.16	17	1.02	0.32	0.31	1.8
De Luca et al., 2008	34	0.56	0.09	0.16	17	1.02	0.32	0.31	1.8
Zoccolotti et al., 2008	29	0.55	0.12	0.22	6	1.31	0.61	0.47	2.4
Paizi et al., 2011	36	0.50	0.09	0.19	18	0.87	0.21	0.24	1.8
Paizi et al., 2013	17	0.48	0.05	0.10	17	0.86	0.19	0.22	1.8
Martelli et al., 2014	43	0.52	0.09	0.17	25	1.14	0.38	0.33	2.2
Unpublished data	19	0.50	0.06	0.12	13	0.71	0.13	0.18	1.4
**Total**	**331**				**172**				
**Mean**		**0.54**	**0.11**	**0.19**		**1.05**	**0.37**	**0.34**	**1.95**

*Mean reading times (seconds per word read) and SDs are reported for typically developing children and children with dyslexia from the listed studies; the size of each group is also reported. The last column reports the dyslexics/controls means ratios.*

As expected, children with dyslexia have higher mean reading times than typically developing readers. On average, their reading times (1.05 s per word) are about twice as slow as those of typically developing children (0.54 s per word). Thus, there is a mean 1.95 ratio between the performance of the two groups (the range of ratios across studies is 1.4–2.4).

Second, dyslexic children are also considerably more variable in their performance. Mean SD is 0.37 in dyslexic children and only 0.11 in typically developing children. Thus, there is co-variance between mean performance and variability, a finding often reported in the RT literature (Wagenmakers and Brown, 2007). Notably, the larger inter-individual variability shown by children with dyslexia goes beyond the proportionality between mean and SD. This is shown by the coefficient of variation values (i.e., the ratio between SD and mean). In all studies the coefficient of variation for dyslexic children is higher than that of typically developing children (mean value = 0.34 for dyslexic children and 0.19 for typically developing children). This finding underscores the difficulty in comparing the two groups through standard parametric analyses. Indeed, these data indicate a strong and systematic violation of the homogeneity assumption, which is critical to apply parametric analyses. These observations are supported by comparisons through the Levene test for equality of variances. In all studies, the test indicated that the variances of the two groups were significantly different (at least, *p* < 0.01).

This large difference in variability points to the inappropriateness of using standard measures of size effects, such as Cohen’s *d* or *eta^2^* which assume homogeneity of variance. In computing *d*, one can use the SD from either sample (as they are assumed to be homogeneous; Cohen, 1988) or, possibly, the mean of the two. However, results would drastically and systematically change if the SD of either group is used. For example, if one computes the Cohen’s *d* value on the first study in **Table 1** (Judica et al., 2002), one obtains very different values depending on which standard deviation is used to calculate *d*. It is 3.64 by using the SD of typically developing children (0.25), 1.10 using the SD of dyslexic children (0.82), and 1.69 using the average between the two SDs. While all these values indicate a large effect it is clear that the estimate of effect size depends heavily upon which SD value is used. In conclusion, standard effect sizes (such as *d* or *eta^2^*) do not appear to capture the main effect of reading deficiency. This is better described as a multiplicative effect. As such, a better descriptor of the effect is provided by the ratio that captures the multiplicative nature of the performance difference between dyslexic and control readers. Clearly, samples from the various studies show different performances. However, the ratios between the performances between the two groups are relatively stable across studies, ranging from 1.4 to 2.4 with an average close to 2.

### Comments

All parametric analyses rest on the homogeneity of variance assumption. Thus, researchers are typically reluctant in abandoning such a basic tenet. A number of data transformations are often adopted to approach normality of distribution and to control for as much as possible of dis-homogeneities of variance. One such example is the log-transformation often used with RTs. In the case of text reading, sometimes time measures (such as s per word) are converted to speed measures (word per s; for a discussion of the advantages and limits of this transformation see Toraldo and Lorusso, 2012). In this perspective, deviations from normality and from homogeneity of variance are seen as accidental perturbations in the data set that need to be corrected for. In contrast, large inter-individual variability is typically associated to developmental/learning phases, and the huge inter-individual variability in DD is an expression of their condition of being still in a early learning phase of reading, whereas at the same age typically developing readers have reached a plateau in their reading performance.

Present data suggest an interesting alternative to the solution of correcting for deviation from homogeneity of variance. Variabilities between the groups are actually truly dis-homogenous as impaired reading is systematically associated to increased individual variability. The prediction of increased SD in DD stems quite clearly from models that aim to account for the presence of global components in the data. For example, within the rate and amount model (RAM) Faust et al. (1999) propose that, when the difference between two groups is accounted for by a global factor, one expects means of different conditions to linearly covariate with the SDs of the corresponding conditions. Further comments on this perspective will be advanced in the section “Group differences in reading: Linear-additive versus multiplicative models” of the Discussion. Throughout the study we will use the ratio between groups’ performance as an index that capture the multiplicative nature of the performance difference between dyslexic and typically developing readers.

### Fluency Differences in Reading Discrete Words

**Table 2A** reports data on single word reading derived from the same studies as in **Table 1**. Mean vocal RTs are reported. Note that different studies used different experimental manipulations, such as length, frequency, morphological structure etc. However, due to our current interest, we report here both data for single conditions and averaged data across conditions.

**Table 2A:** Vocal reaction times (RTs) in several experimental conditions from the listed studies.

	Typically developing children			Children with dyslexia			Groups’ performance ratio	
	Mean	*SD*	Coefficient of variation	*Mean*	*SD*	Coefficient of variation		
**Judica et al., 2002**								
2-letter word	559	57	0.10	742	147	0.20	*1.33*	
3-letter word	574	67	0.12	856	285	0.33	*1.49*	
4-letter word	585	67	0.11	960	433	0.45	*1.64*	
5-letter word	633	87	0.14	1103	535	0.48	*1.74*	
*Mean*	*588*			*915*				*1.55*
**Spinelli et al., 2005**								
3-letter word	639	90	0.14	844	274	0.32	*1.32*	
4-letter word	646	111	0.17	982	479	0.49	*1.52*	
5-letter word	646	107	0.16	1035	435	0.42	*1.60*	
6-letter word	687	191	0.28	1247	565	0.45	*1.81*	
7-letter word	758	173	0.23	1477	657	0.44	*1.95*	
8-letter word	744	209	0.28	1412	643	0.45	*1.90*	
*Mean*	*686*			*1166*				*1.68*
**Barca et al., 2006**								
HF words with contextual rules	685	104	0.15	850	95	0.11	*1.24*	
HF words without contextual rules	675	105	0.16	862	92	0.11	*1.28*	
LF words with contextual rules	768	126	0.16	1000	153	0.15	*1.30*	
LF words without contextual rules	725	127	0.18	955	134	0.14	*1.32*	
*Mean*	*713*			*917*				*1.28*
**Zoccolotti et al., 2006**								
4-letter word	708	93	0.13	997	207	0.21	*1.41*	
5-letter word	716	105	0.15	1088	222	0.20	*1.52*	
6-letter word	745	137	0.18	1306	392	0.30	*1.75*	
7-letter word	784	138	0.17	1371	523	0.38	*1.75*	
*Mean*	*738*			*1190*				*1.61*
**Burani et al., 2008**								
Derived words (Exp2)	672	114	0.17	1086	485	0.45	*1.62*	
Simple words	670	108	0.16	1103	487	0.44	*1.65*	
*Mean*	*671*			*1095*				*1.63*
**De Luca et al., 2008**								
4-letter word	612	63	0.10	816	246	0.30	*1.33*	
5-letter word	624	74	0.12	886	294	0.33	*1.42*	
6-letter word	622	70	0.11	912	305	0.33	*1.47*	
7-letter word	649	90	0.14	1039	441	0.42	*1.60*	
8-letter word	691	103	0.15	1110	480	0.43	*1.61*	
9-letter word	710	104	0.15	1153	496	0.43	*1.62*	
*Mean*	*651*			*986*				*1.51*
**Zoccolotti et al., 2008**								
4-letter word	601	67	0.11	851	297	0.35	*1.42*	
5-letter word	612	65	0.11	803	221	0.27	*1.31*	
6-letter word	599	72	0.12	983	420	0.43	*1.64*	
7-letter word	655	82	0.12	1012	332	0.33	*1.55*	
8-letter word	611	82	0.13	1047	641	0.61	*1.71*	
*Mean*	*616*			*939*				*1.53*
**Paizi et al., 2011**								
HF 4-letter word - pure list	540	57	0.10	685	137	0.20	*1.27*	
HF 5-letter word - pure list	553	62	0.11	713	127	0.18	*1.29*	
HF 6-letter word - pure list	566	62	0.11	779	165	0.21	*1.38*	
HF 7-letter word - pure list	558	69	0.12	783	165	0.21	*1.40*	
LF 4-letter word - pure list	555	60	0.11	732	134	0.18	*1.32*	
LF 5-letter word - pure list	568	69	0.12	807	165	0.20	*1.42*	
LF 6-letter word - pure list	579	69	0.12	843	205	0.24	*1.46*	
LF 7-letter word - pure list	595	83	0.14	974	328	0.34	*1.64*	
HF 4-letter word - mixed list	563	57	0.10	702	111	0.16	*1.25*	
HF 5-letter word - mixed list	582	62	0.11	786	174	0.22	*1.35*	
HF 6-letter word - mixed list	601	74	0.12	842	199	0.23	*1.40*	
HF 7-letter word - mixed list	590	72	0.12	817	174	0.21	*1.39*	
LF 4-letter word - mixed list	587	64	0.11	753	149	0.20	*1.28*	
LF 5-letter word - mixed list	598	67	0.11	798	139	0.17	*1.33*	
LF 6-letter word - mixed list	610	79	0.13	876	222	0.25	*1.44*	
LF 7-letter word - mixed list	626	88	0.14	956	265	0.28	*1.53*	
*Mean*	*580*			*803*				*1.38*
**Paizi et al., 2013**								
HF word (Exp.1)	597	50	0.08	794	72	0.09	*1.33*	
LF word (Exp.1)	632	53	0.08	898	150	0.17	*1.42*	
HF 4-letter word (Exp.3)	510	78	0.15	679	98	0.14	*1.33*	
HF 5-letter word (Exp.3)	534	74	0.14	741	106	0.14	*1.39*	
HF 6-letter word (Exp.3)	543	86	0.16	812	212	0.26	*1.49*	
HF 7-letter word (Exp.3)	539	78	0.14	808	167	0.21	*1.50*	
LF 4-letter word (Exp.3)	544	80	0.15	753	102	0.13	*1.38*	
LF 5-letter word (Exp.3)	568	88	0.15	833	166	0.20	*1.47*	
LF 6-letter word (Exp.3)	568	97	0.17	864	192	0.22	*1.52*	
LF 7-letter word (Exp.3)	580	94	0.16	959	311	0.32	*1.65*	
*Mean*	*562*			*814*				*1.45*
**Martelli et al., 2014**								
4-letter word	506	59	0.12	827	361	0.44	*1.63*	
5-letter word	511	69	0.13	939	433	0.46	*1.84*	
6-letter word	536	76	0.14	1056	502	0.47	*1.97*	
7-letter word	541	95	0.17	1150	585	0.51	*2.13*	
*Mean*	*524*			*993*				*1.89*
**Unpublished data**								
HF 4-letter word	490	40	0.08	547	58	0.10	*1.12*	
HF 5-letter word	480	40	0.08	568	72	0.13	*1.18*	
HF 6-letter word	491	45	0.09	594	84	0.14	*1.21*	
HF 7-letter word	497	43	0.09	589	88	0.15	*1.18*	
LF 4-letter word	496	42	0.08	564	56	0.10	*1.14*	
LF 5-letter word	507	38	0.07	594	66	0.11	*1.17*	
LF 6-letter word	509	40	0.08	611	77	0.13	*1.20*	
LF 7-letter word	514	50	0.10	613	67	0.11	*1.19*	
*Mean*	*498*			*585*				*1.17*
**Total mean^∗^**	**602.5**	**82.6**	**0.13**	**891.7**	**265.3**	**0.28**	**1.47**	**1.52**
***SD***	**75.2**			**202.9**				

*Mean vocal RTs (milliseconds per word), standard deviations and coefficients of variation for typically developing children and children with dyslexia are reported. The last column reports the dyslexics/controls mean ratios. ^∗^Total means do not include partial means.*

An inspection of the table indicates a number of relevant findings. Clearly, children with dyslexia are slower than typically developing children across conditions. All studies in this re-analysis, showed a highly significant main effect of the group factor (with at least *p* < 0.01) at standard Anovas. However, the ratios between the two groups are consistently lower than those in **Table 1**. Across studies and experimental manipulations the overall mean ratio is 1.52 (range across experiments from 1.28 to 1.89; range across all experimental manipulations 1.12 and 2.13); this mean value is considerably lower than that for text reading (1.95, see **Table 1**). Thus, the slowing of dyslexic children with respect to typically developing readers is about 95% in text reading and only 52% in the case of single word reading. If, instead of averaged data, we separately compare the between groups’ ratios for each of the 69 experimental conditions, in only two cases are the ratios above the mean value (1.95) obtained for text reading (see **Table 2A**).

Notably, values vary across experimental manipulations. In particular, in studies manipulating length (as in the first one by Judica et al., 2002) there is a clear tendency for ratios to increase as a function of stimulus length (in this case from 1.33 to 1.74 with progressively longer words). The same is apparent in most (Spinelli et al., 2005; Zoccolotti et al., 2006, 2008; De Luca et al., 2008; Paizi et al., 2011, 2013; Martelli et al., 2014) although not all (research with unpublished data) studies. The other variable that has been manipulated most often is frequency. Across all contrasts between high and low frequency words, the ratios between the two groups for the high frequency words averaged 1.31 while those for the low frequency words averaged 1.38. Thus, ratios do not vary appreciably between conditions as a function of frequency. It may be interesting to compare these findings to the calculations based on more sophisticated methods, such as the analyses based on the RAM by Faust et al. (1999) which were carried out in several of the quoted studies. This may help understanding the efficacy, and limits, of the procedure of using the ratio as an estimate of size of the group differences in reading skills; further comments on this question will be proposed in the Discussion section.

Dyslexic children are considerably more variable as a group than typically developing children; their average SD is 265.3 ms while that of control readers is only 82.6 ms. In general, variability grows as a function of the general difficulty of the experimental conditions with more difficult conditions yielding larger SD. Across conditions there is a 0.81 correlation (*p* < 0.001) between means and SDs in control children; the correlation is 0.86 (*p* < 0.001) for dyslexic children. These results are in keeping with the general law indicating a relationship between condition means and standard deviations for RT measures (Wagenmakers and Brown, 2007). Furthermore, also coefficients of variation are about twice as high in dyslexic children (mean value = 0.28) than in control readers (mean value = 0.13); for only 6 out 69 conditions were the coefficients of variation higher for control than dyslexic readers. Comparisons with the Levene test indicated that the variances of the two groups were significantly different (with at least *p* < 0.05) in 53 out of 69 comparisons.

### Comments

Despite variations across studies and experimental conditions, the ratio data clearly indicate that vocal RTs of dyslexic children are slower than typically developing readers by about 50%. This contrasts with the ratios measured for reading times, where dyslexic children were about 100% slower than typically developing children.

### From RTs to Total Reading Times in Reading Discrete Words

One general finding of the above analyses is that ratios between the performance of dyslexic and typically developing readers in the case of multiple stimulus displays are higher than in the case of discrete stimulus displays. Clearly, the two sets of data refer to different measures. In the case of discrete stimuli only the time between stimulus presentation and the *incipit* of the response is considered but not the actual pronunciation time. By contrast, in the case of multiple stimulus displays the measure is the total reading time (i.e., it includes pronunciation time). So, one may consider how the use of different measures affect the results.

A way to tackle this problem is to include pronunciation time measures in experiments with single stimulus displays. RTs and pronunciation times together give a measure of total reading time, which may be usefully compared to the mean reading time per item in the case of multiple stimulus displays. Measuring pronunciation times is simple although time consuming as it requires trial-by-trial analysis. A few studies have used this procedure in recent times (e.g., Davies et al., 2013). One of the studies in **Table 2A** also adopted this procedure (Martelli et al., 2014).

Martelli et al.s’ (2014) results for pronunciation times and total reading times (i.e., RTs plus pronunciation times) are presented in **Table 2B** and can be compared with RT data for the same study presented in the low part of **Table 2A**. Across conditions the ratio between the performance of dyslexic and control readers is 1.89 for RTs in this particular study (i.e., a value in the high range compared to similar studies in the same table). The ratio (see **Table 2B**) is close to unity in the case of pronunciation times (1.04); thus, across conditions children with dyslexia show pronunciation times very similar to those of control children and also very similar inter-individual variability (as indicated by both SDs and coefficients of variations). When considering total reading times, the groups’ performance ratio is 1.48, i.e., intermediate between those obtained with the two measures contributing to total reading time (i.e., vocal RTs and pronunciation). In particular, this value is much smaller than the one obtained in the same study in the case of RTs (1.89; see **Table 2A**).

**Table 2B:** Pronunciation times and total reading times from Martelli et al.’s (2014) study.

	Typically developing children			Children with dyslexia			Groups’ performance ratio	
	Mean	*SD*	Coefficient of variation	Mean	*SD*	Coefficient of variation		
**Pronunciation time**								
4-letter word	395	60	0.15	404	51	0.13	*1.02*	
5-letter word	461	59	0.13	468	69	0.15	*1.02*	
6-letter word	487	63	0.13	510	71	0.14	*1.05*	
7-letter word	564	75	0.13	596	83	0.14	*1.06*	
*Mean*	*477*	*64*	*0.14*	*494*	*68.5*	*0.14*		*1.04*
**Total reading time**								
4-letter word	901	89	0.10	1231	366	0.30	*1.37*	
5-letter word	972	103	0.10	1406	455	0.32	*1.45*	
6-letter word	1023	101	0.10	1565	522	0.33	*1.53*	
7-letter word	1106	127	0.11	1746	611	0.35	*1.58*	
*Mean*	*1000*	*105*	*0.10*	*1487*	*489*	*0.33*		*1.48*

*Mean pronunciation time and mean total reading time (milliseconds per single word), standard deviations and coefficients of variation are reported for typically developing children and children with dyslexia. The last column reports the dyslexics/controls mean ratio.*

We can use the values measured in this study to estimate the average drop of the mean ratio in the case of total reading time as opposed to vocal RTs to discrete words. The proportion

1.52:x = 1.89:1.48

where 1.52 is the mean ratio for RTs across studies; and the two remaining values are the ratios for RTs and total reading time in the Martelli et al.’s (2014), study, respectively. The proportion leads to an estimated groups’ ratio of 1.19 when total reading time of single words is considered. As this is based on a single study this is clearly a rough estimate of the groups’ ratio for discrete word presentation. However, it generally indicates that the difference in groups’ ratios between multiple (1.95) and discrete (1.52) displays is likely underestimated by the use of RTs rather than total reading times and is presumably much larger.

### Comments

Overall, the results indicate that the RT groups’ ratios are presumably a high estimate of the groups’ differences in single word reading, as RTs are only the part of the response that is most sensitive to the experimental manipulations. If one includes also the component of pronunciation, which distinguishes minimally between the two groups, the ratios drop substantially indicating that the differences in groups’ ratios between multiple and discrete displays are much larger than those estimated based on overall text reading on the one side and vocal RTs to words (as in **Table 2**) on the other. Indeed, the present computations indicate a group ratio of 1.95 in the case of multiple displays (see **Table 1**) and an overall estimate of 1.19 in the case of discrete displays (according to the formula above); this is a quite large difference in size effect. If confirmed by subsequent studies (it would be interesting that future studies also consider total reading times in RTs experiments), this pattern of findings would indicate that efficiency in reading aloud single words plays only a moderate role in determining the fluency of dyslexic children when reading texts, which would certainly be a surprising finding. An important, and generally neglected role would be played by the other components involved in the reading task.

### Reading Lists of Words

One additional confounding factor when comparing reading texts with reading isolated words is the presence of contextual information only in the former, but not the latter, case. So, the larger group differences in text reading may depend upon a selective difficulty of dyslexic children to integrate the semantic context. Indeed, there is reason to consider this hypothesis unlikely. Children with dyslexia do not show a selective deficit in comprehending texts at least in the case in which no time limit is imposed, as in the standard procedure of the *MT Reading Test* (Cornoldi and Colpo, 1995). Typically, under these conditions, dyslexic children show only a mild defect or even an entirely spared performance (e.g., Zoccolotti et al., 1999). Still, one could envisage the hypothesis that, at least under conditions in which both speed and accuracy are encouraged (as it is required to the children in the standard *MT Reading Test*), the need for an ongoing integration of successive pieces of information may provide an additional burden widening the performance difference between the two groups.

Information on this question may come from conditions in which the child is asked to read lists of unrelated words printed on a page. Under these conditions, no role of context is present and no need to integrate the meaning of successive information is required for effective performance. In some of the studies listed in **Table 1** we also used two such tests (*Words and Non-words Reading Test*; Zoccolotti et al., 2005, and the word list from the *Battery for the Evaluation of* DD *and Dysgraphia*; Sartori et al., 1995). In the former test four separate measures are taken for words varying for frequency and length; for the latter test a single measure is usually reported (based on available norms).

For four studies, there are data on the *Words and Non-words Reading Test* (see **Table 3A**). Across studies and conditions there is a ratio of 1.83 (range 1.51–2.26) between the performance of dyslexic and control readers. This estimate is lower than the one observed in the case of text reading (1.95) but higher than the one for single word reading (1.52) particularly if one considers the need for a correction due to the use of RTs rather than total reading times. On average, the ratios are slightly higher for low (1.89) than high (1.77) frequency words, and higher for long (1.96) than short (1.71) words. As in previous comparisons, dyslexic children were more variable than typically developing children, both in terms of SDs (0.74 vs. 0.24, respectively) and of coefficients of variation (0.47 vs. 0.27, respectively). Comparisons with the Levene test indicated that the variances of the two groups were significantly different (with at least *p* < 0.05) in 14 out of 16 comparisons.

**Table 3A:** Reading times (s per word) for the Words and Non-words Reading Test (Zoccolotti et al., 2005) from the listed studies.

	Typically developing children			Children with dyslexia			Groups’ performance ratio	
	Mean	*SD*	Coefficient of variation	Mean	*SD*	Coefficient of variation		
**Spinelli et al., 2005**								
4-to-5-letter high frequency words	0.76	0.21	0.28	0.97	0.50	0.52	*1.27*	
8-to-10-letter high frequency words	0.99	0.25	0.25	1.62	0.78	0.48	*1.63*	
4-to-5-letter low frequency words	0.86	0.25	0.29	1.25	0.73	0.59	*1.46*	
8-to-10-letter low frequency words	1.38	0.47	0.34	2.30	0.90	0.39	*1.66*	
*Mean*	*1.00*			*1.53*				*1.51*
**Zoccolotti et al., 2006**								
4-to-5-letter high frequency words	0.61	0.18	0.29	0.98	0.43	0.44	*1.61*	
8-to-10-letter high frequency words	0.78	0.20	0.25	1.61	0.82	0.51	*2.06*	
4-to-5-letter low frequency words	0.69	0.19	0.28	1.25	0.58	0.46	*1.82*	
8-to-10-letter low frequency words	1.09	0.31	0.28	2.31	0.96	0.41	*2.13*	
*Mean*	*0.79*			*1.54*				*1.91*
**Burani et al., 2008**								
4-to-5-letter high frequency words	0.68	0.20	0.29	1.04	0.41	0.40	*1.53*	
8-to-10-letter high frequency words	0.87	0.22	0.25	1.45	0.62	0.43	*1.67*	
4-to-5-letter low frequency words	0.76	0.22	0.28	1.33	0.48	0.36	*1.74*	
8-to-10-letter low frequency words	1.21	0.34	0.28	2.03	0.61	0.30	*1.68*	
*Mean*	*0.88*			*1.46*				*1.66*
**Zoccolotti et al., 2008**								
4-to-5-letter high frequency words	0.62	0.13	0.21	1.21	0.76	0.62	*1.96*	
8-to-10-letter high frequency words	0.80	0.15	0.19	1.96	1.08	0.55	*2.43*	
4-to-5-letter low frequency words	0.73	0.17	0.23	1.66	1.10	0.66	*2.29*	
8-to-10-letter low frequency words	1.18	0.34	0.29	2.82	1.15	0.41	*2.38*	
*Mean*	*0.83*			*1.91*				*2.26*
**Total mean^∗^**	**0.87**	**0.24**	**0.27**	**1.61**	**0.74**	**0.47**	**1.83**	**1.83**
***SD***	**0.23**			**0.54**				

*Means, standard deviations, and coefficients of variation for typically developing children and children with dyslexia are reported. The last column reports the dyslexics/controls mean ratio. ^∗^Total means do not include partial means.*

As to the word list from the from the *Battery for the Evaluation of* DD *and Dysgraphia* (Sartori et al., 1995) there are data available from two of the studies with information on discrete word reading (see **Table 3B**). In all studies the ratio between the performance of dyslexic and control readers was above 2 (mean = 2.62), a value higher than that in the case of text reading.

**Table 3B:** Reading times (s per word) for the word list from the Battery for the Evaluation of Developmental Dyslexia (DD) and Dysgraphia (Sartori et al., 1995) from the listed studies.

	Typically developing children			Children with dyslexia			Groups’ performance ratio	
	Mean	*SD*	Coefficient of variation	Mean	*SD*	Coefficient of variation		
Judica et al., 2002	0.74	0.30	0.41	1.81	0.81	0.45	2.44	
Spinelli et al., 2005	0.74	0.30	0.41	2.07	0.90	0.43	2.80	
***Mean***	**0.74**	**0.30**	**0.41**	**1.94**	**0.86**	**0.44**	**2.62**	
***SD***	**0.00**			**0.19**				

*Means, standard deviations, and coefficients of variation for typically developing children and children with dyslexia are reported. The last column reports the dyslexics/controls mean ratio.*

### Comments

The data available in the case of word lists are fewer than those on text reading and the results are also somewhat scattered with higher ratios for the word list from the *Battery for the Evaluation of* DD *and Dysgraphia* (Sartori et al., 1995) than for the word lists from the *Words and Non-words Reading Test* (Zoccolotti et al., 2005). Differences in list composition probably account for this effect although it is at present difficult to understand which feature in the list composition is critical to yield such outcome. However, data from word lists are generally in keeping with the idea that reading multiple words generates greater group differences than reading discrete words. This occurs in the absence of any contextual effect. Thus, it appears that the requirement to read a sequence of stimuli rather than a single one is sufficient to generate a large size group difference also in the absence of a meaningful semantic context.

It should be added that these data do not allow excluding the possibility that the context exerts some at least partial effect in modulating the group differences in reading fluency. To obtain a definite response on this point would require stimuli which vary only along the context dimension; e.g., comparing regular and scrambled matched texts may be instrumental to clarify this question. In this respect, it should be noted that the possible direction of such an effect is not obvious. On the one side, one could envisage that, since they have generally spared semantic skills, dyslexic children may actually be favored by the presence of contextual information. On the other, one could hypothesize that in a time demanding task the need to online process the information concerning the syntactic relationship between words represents an additional burden, which further dampens performance. *Ad hoc* research is needed to clarify this point. However, the present data seem sufficiently clear to indicate that the need to process multiple stimuli poses by itself a selective stress on dyslexic children such that their difference in performance with control readers becomes much more pronounced than that observed in the case of discrete displays.

## Discussion

Comparing the performance of dyslexic and typically developing readers in tasks such as reading texts, lists of words and single words poses challenging methodological questions and the present data only represent an initial sketch of the complex set of relationships that may influence reading fluency. Furthermore, it seems important that the present data should be supported by additional evidence from other research groups. However, even the available evidence seems strong enough to conclude that, at least for Italian language, reading multiple stimuli present a specific challenge to the dyslexic children at the sixth grade of schooling indicating that models of reading should incorporate this aspect (e.g., Zoccolotti et al., 2014). By contrast, up to date most models of reading are based on the assumption that the reading process can be explained at the single word level (Plaut et al., 1996; Coltheart et al., 2001; Perry et al., 2007).

The reviewed data seem sufficiently persuasive to conclude that group differences (dyslexic vs. typically developing readers) in reading fluency in the case of multiple word displays are much greater than differences in the case of discrete word displays. In fact, as shown above, the difference between the two sets of data are presumably larger than they appear based on available data. In the case of discrete stimulus presentations, typically RTs are presented; this measure extracts the portion of the response that is most sensitive to the decoding differences. However, if one considers a measure (total reading time) that is more similar to that used in text or words list reading, a much greater difference emerges between discrete and multiple displays.

### Deficits in Multiple Displays

Clear differences in reading isolated words are present between typically developing and dyslexic readers. However, dyslexic readers have larger deficits compared to typically developing readers when they have to deal with multiple displays. Reading in these conditions requires integration of various sub-components. While processing the ongoing word, the reader has to perform some parafoveal analysis of the next word, to program the more effective landing of the next forward saccade (often skipping functional words; for a review see Rayner, 2009). The output of word processing is held in memory in order to effectively synchronize the pronunciation of the stimulus with the decoding of the subsequent words (referred to as eye-voice lead; Fairbanks, 1937). Reading under these conditions selectively dampens dyslexic performance. Thus, it appears that, in understanding the reading impairment of dyslexic children, one has also to explain this failure with multiple stimuli and not limit the interpretation to the deficit at a single-word level.

Why should dyslexic children be selectively impaired in dealing with multiple visual displays? One can envisage four possible scenarios.

Firstly, one could consider the text reading deficit as a cascade effect of the nuclear defect in orthographic decoding. The deficit might be amplified through the greater complexity, and henceforth difficulty, involved in text reading. According to this view, even if the reading deficits for discrete and multiple displays have different sizes (the latter being greater than the former), they would essentially refer to the same deranged mechanism. Within this hypothesis, the deficit with discrete displays should accurately predict the one with multiple displays. By contrast, there is evidence that, in accounting for individual differences in text reading fluency, the performance on rapid automatized naming (RAN) tasks (Denckla and Rudel, 1974) increases the variance explained by single word reading in Greek (Protopapas et al., 2013) and Italian (Zoccolotti et al., 2014) readers. This finding is not in keeping with the idea that a single deficit explains impairments with discrete and multiple displays.

Second, it is conceivable that, in addition to the decoding deficit (which is clearly evident also in the present re-analysis), dyslexic children have a selective deficit in one of the other reading subcomponents. While it is likely that at least some of the children may have additional defects, previous attempts along this line have been generally unsuccessful. For example, as shown above, articulation deficits are absent (e.g., Martelli et al., 2014). As for a deficit in the programming and execution of eye movements as suggested in an early study (Pavlidis, 1981), most successive evidence has been inconsistent with this hypothesis (e.g., Brown et al., 1983; Olson et al., 1983; De Luca et al., 1999); i.e., dyslexic children have eye movements comparable to controls except when dealing with reading material. Further, in spite of their deranged pattern of eye movements during reading, impaired readers show an intact mechanism for performing corrective re-fixations (a mechanism linked to oculomotor and visual processes not linguistic ones; Gagl et al., 2014). Although some researchers are still working on the hypothesis that some selective deficits in eye movements programming or execution may actually be impaired in dyslexic children (e.g., Bucci et al., 2008) this hypothesis seems poorly supported by evidence. Overall, the available results do not seem strong enough to account for the large differences in text reading fluency although it is difficult to reach definite conclusions on this literature.

A third scenario is to focus on the possible interaction of the various sub-components underlying multiple word reading with the reading deficit. Even though none of the sub-components (apart from orthographic decoding) reveals a selective deficit (as envisaged in the case of the second scenario), the presence of a deficit in orthographic decoding could make the multi-task management considerably more difficult (De Luca et al., 2013). For example, in this view, dyslexic children would not be impaired in parafoveal processing *per se*. However, the need to process the next (right) word parafoveally to appropriately calibrate the successive saccade may be hindered by the attention of the child being fully focused on the ongoing target word in the troubled attempt to process it. There is some evidence supporting this view (Yan et al., 2013). Overall, one could posit that a set of processes, which are in themselves spared, represent an attention overload due to the presence of a selective deficit in orthographic decoding. In this interactive view, orthographic decoding would indirectly dampen text reading fluency as it may prove difficult to carry out a complex task if one does not manage well one of the task sub-components (De Luca et al., 2013). This third scenario does not require any additional deficit (as in the second scenario) or amplification (as in the “cascade” first scenario) other than the defect in orthographic decoding. However, one may imagine that factors, such as divided attention, may interact with the decoding deficit in modulating the reading fluency of children with dyslexia. According to this interactive view (and differently from the cascade view) one would not expect the single-word decoding deficit to accurately predict the deficit with multiple words. Furthermore, one would not expect performance on divided attention tasks and/or executive tasks to directly correlate with reading. However, one could put forward the hypothesis that performance on these tasks may act as suppressor variables allowing for increased prediction in the case of reading words in multiple (but not single) displays. Communality analyses may allow the detection of such suppression effects. Overall, integrating several sub-components of the reading task may pose an additional, partially independent, challenge to the dyslexic children (Zoccolotti et al., 2014).

A fourth scenario to explain the greater fluency deficit of dyslexic children with multiple than single word displays focuses on the difference between the experimental conditions used in the two sets of tasks. In the single condition, the word is abruptly displayed on the screen; in the multiple conditions, the words are statically displayed on a sheet of paper (or a PC screen; the support does not probably make a critical difference). It is well known that the abrupt onset of a stimulus is perceptually salient, captures bottom-up attention (Jonides and Yantis, 1988), elicits prepotent and fast saccades (McDowell et al., 2008), and triggers fast visual processing up to target identification (indicated by shorter RTs in search tasks; e.g., Theeuwes, 1994) or word decoding (indicated by reading rate increment in Rapid Serial Visual Presentation task; Rubin and Turano, 1992). By contrast, reading in the static condition of a multiple display implies a more internally driven visual scanning of the items; saccades (and decoding) are self-paced and driven by parafoveal pre-analysis (Schotter et al., 2012). It is likely that these differences between static and dynamic reading conditions are relevant for the overall speed of processing. Indeed, the neural network involved in self-paced and externally triggered movements do not entirely overlap and have different time constants (Thickbroom and Mastaglia, 1985; Cunnington et al., 2002). Consistently, some recent EEG (Dimigen et al., 2011, 2012) and fMRI (Choi et al., 2014; Richlan et al., 2014) studies investigating the neural basis of reading have privileged the ecological method of sentence reading rather than single-word reading or rapid serial visual presentation. In this perspective, single word presentation may facilitate reading processing by automatic recruitment of attention and by providing an external pacing of the reading activity; this facilitation might be particularly advantageous (in terms of speed) for dyslexic children with respect to typically developing readers. Some authors described the “sluggish” attention (Hari and Renvall, 2001) of dyslexic children. This defect would be partially overcome by abrupt presentation of stimuli. In other terms, an externally triggered onset of the target word would make the reading of dyslexic children more “automatized”, that is, more similar to the reading of typically developing readers. Consequently, the difference between groups would be less marked in the case of single stimulus displays. The very high correlation between text reading and individual speed in RAN tasks (where multiple color patches or objects have to be named) but not in single color naming (when the color patch is abruptly displayed on the screen) may be seen as supporting this line of interpretation (Georgiou et al., 2013). To test this hypothesis, it may prove instrumental to compare reading of multiple word displays in conditions in which the observer is requested to read words at his/her own self-pace or some external abrupt cue (such as a bar underlining the target word) introduces an imperative stimulus in the display. Reading under externally paced conditions is expected to yield smaller group differences between dyslexic and control readers.

The present evidence is still too sparse to definitively choose among these alternatives. However, some facts seem clear. In particular, the lack of a strong correlation between performance on discrete and multiple displays (de Jong, 2011) is inconsistent with the first “cascade” scenario. Also, the search for selective deficits in eye movements programming and execution has yet proven unsuccessful making also the second scenario unlikely. However, the last two scenarios seem promising venues for future research; some possible hypotheses worth testing have been outlined.

### Group Differences in Reading: Linear-Additive versus Multiplicative Models

As compared to typically developing children, dyslexic readers are not only much slower but also considerably more variable in their performance. This is indicated by much greater SDs and coefficients of variation. Thus, greater variability goes even beyond what might be anticipated on the basis of an increase in the mean performance. Multiplicative models may account for this pattern more effectively than linear additive models.

One such model is the RAM proposed by Faust et al. (1999). Accordingly, performance depends multiplicatively by an individual factor (the rate at which the individual processes information) and by a task related factor (the difficulty of the given experimental condition referred to as “amount”). Along this reasoning, performance on a given condition does not merely express the specific ability to deal with a given specific condition but also depends upon more general factors such as the global ability of the individual to process information and the general difficulty of the task (over and above the specificity of the experimental condition). Note that this perspective generally indicates a situation often referred to as “task impurity”: i.e., there is a lot more in the performance in any given task than the specific process which is intended to probe. To express the rate factor in DD we have referred to a global factor in orthographic pre-lexical processing. In this view, individuals have a characteristic speed in processing orthographic materials which influences all conditions and tasks which require to visually process orthographic strings of letters. So, this factor is global in the sense that is not condition-specific but it affects all conditions within the orthographic domain (such as naming long and short words, high and low frequency words, naming non-words, lexical decision). However, it is not to be intended as “general” as it does not apply to task in which other types of stimuli are to be processed (e.g., naming objects; Zoccolotti et al., 2008) or word stimuli are to be processed in a sensory modality different from the visual one (i.e., with auditory presentation; Marinelli et al., 2011).

The present analyses indicate that the multiplicative nature of the difference between dyslexic and typically developing readers is well captured by ratios while it is not well accounted for by effect size measures (such as Cohen’s *d*) within the parametric linear additive perspective. In this context, ratios present advantages but also limitations. The main advantage is that they allow to quickly compare performances in otherwise disparate conditions, which would be difficult to compare within the rather selective requirements of models which aim to account for individual differences in performance in timely tasks. Here, we showed that the ratios for reading performance in the case of multiple visual displays are considerably higher than those for reading performance in the case of single visual displays.

An important limitation of using the ratio is that this value indicates an overall relationship between the performances of the two groups. By contrast, an attempt of models such as the RAM, the DEM or the diffusion model is to distinguish between different components of the response. So, according to Myerson et al. (2003) one could separate a decisional and a non-decisional part of the response (and clear predictions are put forward to tease out these two components of the response). Based on these predictions, Martelli et al. (2014) showed that it was only the decisional component of the response which contributed in generating the group differences in performance. Using a lexical decision, a similar conclusion was reached by Zeguers et al. (2011) who, based on a diffusion model analysis, observed no difference between dyslexic and control readers in the non-decision components of the RTs. Indeed, the diffusion model makes a step ahead and, beyond the distinction between decisional and non-decisional components, is also able to account for the possible modulating role of criterion (or “conservatoriness”) in mediating the group differences (Ratcliff, 1978). However, also in this case, experimental conditions are constrained within rather strict requirements and it is not immediately apparent how group differences in multiple versus single stimulus displays could be examined within the experimental requirements envisaged by these models.

Empirically, it may be instructive to examine whether ratios capture effects in ways which are more or less compatible with the more tuned analyses performed in relationship with the above mentioned models. To test the possible presence of selective effects over and beyond the effect of the global factor in orthographic processing in a number of studies we referred to the RAM (Faust et al., 1999). This proposes a number of data transformations (including an individually based *z*-score transformation) which allow obtaining condition measures stripped off the effect of the global factor^1^. This transformation allows distinguishing between group by condition interactions which can be entirely ascribed to an over-additivity effect and those in which a residual, selective effect of a specific experimental variable is detectable. In several experiments we found that, if one examines raw RT data, dyslexic children show larger frequency effects than control readers. However, if one controls for the effect of the global factor by normalizing data over individual subjects as suggested by Faust et al. (1999), the group by frequency interaction disappears (Paizi et al., 2013). By contrast, in a number of studies we found that the effect of stimulus length was detected even after accounting for the effect of the global factor (e.g, Zoccolotti et al., 2008).

When we re-examine the results of these experiments by using ratios it is clear that frequency plays no detectable role in the case of RT studies (see **Table 2**) and a very limited role in the case of total reading times (**Table 3A**). By contrast, length exerts a very clear impact on ratio values in the case of RT data (**Table 2**) and some influence also in the case of total reading times (**Table 3A**). Therefore, it appears that, although results in terms of ratios represent less sophisticated measures of group differences than those that may be obtained with reference to models such as the RAM or DEM they yield a pattern of results which is generally consistent with that obtained with reference to these models. This reinforces the idea that the large difference in ratios between discrete and multiple displays is a genuine phenomenon, not one derived from the adoption of such a measure.

In conclusion, the idea that group differences in reading do not easily fit with linear additive models has indeed widespread implications. Nearly all the literature on reading skills uses parametric analyses based on linear additive assumptions. When deviations from normality are detected, appropriate data transformations (such as log transform in the case of RTs or speed, as opposed to time, measures in the case of texts) are used. Furthermore, it is generally held that results from ANOVAs are generally quite robust, in that they are not very sensitive to deviations from normality. So, we certainly do not wish to claim that all results in the literature are faulty or unreliable. Rather, we would like to make the general point that it seems unfounded to try to explain by means of linear additive models differences which are clearly multiplicative. If seen within a linear additive model group differences are prone to be sensitive to over-additivity effects, i.e., more difficult conditions will generate larger group differences over and above the influence of a specific experimental manipulation. By contrast, examining the group differences from the perspective of multiplicative models (such as the RAM) may potentially allow separating the different factors that contribute in generating individual differences in performance.

## Conclusion

Children with dyslexia show a clear impairment in reading words when they are singly presented (and vocal RTs are measured). In particular, they are both slower and considerably more variable than typically developing readers. This pattern of results is consistent with the idea that the deficit is best expressed in terms of a multiplicative rather than additive difference. Thus, an effective way to describe the group difference is with the use of ratios rather than standard measures of size effects (such as Cohen’s *d*).

The RT measure is very sensitive to capture the part of the response most sensitive to the reading deficit. Thus, the very clear results obtained measuring RTs to single word presentation may give the impression that the reading deficit is strong and independent of the number of targets present in the display. However, if care is taken in using appropriate comparisons, it is clear that the deficit in reading texts or lists of words is appreciably greater than that revealed with discrete stimulus presentations. Thus, to fully explain the reading deficit of these children one should also account for their difficulty in managing the complex set of sub-component tasks underlying the fluent read a text. While several hypotheses can be put forward to explain this deficit, the present re-analysis underscores that an exhaustive explanation of the reading deficit cannot be obtained based on the performance on single word presentations only.

## Conflict of Interest Statement

The authors declare that the research was conducted in the absence of any commercial or financial relationships that could be construed as a potential conflict of interest.

## Footnotes

1 The formal limits of using a ratio (or proportion) transformation have been discussed by Faust et al. (1999). Essentially, as this transformation identifies an overall relationship between two measures the results would be identical to those of using transformations, such as the *z* score or regression transformations, only in the case in which the additive constant (i.e., the intercept) of the relationship is null.
